# Ovarian adrenal rest tumor in congenital adrenal hyperplasia: Is medical treatment the first line option?

**DOI:** 10.20945/2359-3997000000415

**Published:** 2021-11-11

**Authors:** Ronit Koren, Shlomit Koren, Alla Khashper, Carlos Benbassat, Marina Pekar-Zlotin, Zvi Vaknin

**Affiliations:** 1 Shamir Medical Center Department of Internal Medicine A Zerifin Israel Department of Internal Medicine A, Shamir Medical Center, Zerifin, Israel; 2 Tel-Aviv University Sackler Faculty of Medicine Tel-Aviv Israel Sackler Faculty of Medicine, Tel-Aviv University Tel-Aviv, Israel; 3 Shamir Medical Center Endocrine Institute Zerifin Israel Endocrine Institute, Shamir Medical Center, Zerifin, Israel; 4 Shamir Medical Center Diabetes Unit Zerifin Israel Diabetes Unit, Shamir Medical Center, Zerifin, Israel; 5 Shamir Medical Center Department of Diagnostic Imaging Zerifin Israel Department of Diagnostic Imaging, Shamir Medical Center, Zerifin, Israel; 6 Shamir Medical Center Department of Obstetrics and Gynecology Zerifin Israel Department of Obstetrics and Gynecology, Shamir Medical Center, Zerifin, Israel

## Abstract

Ovarian adrenal rest tumors (OARTs) are very rare. We describe a case of a young woman with uncontrolled classical congenital adrenal hyperplasia (CCAH), presenting with bilateral OARTs, successfully treated with steroid replacement. A 20-year-old woman, known to have 21OH-CCAH, presented with severe abdominal pain, vomiting, diarrhea, and fever. As a result of poor compliance, 6 months before her admission hirsutism worsened and amenorrhea, hyperpigmentation, and weakness developed. ACTH levels were 278 < pmol/L and 17OHP 91.3 nmol/L. She was admitted for parenteral antibiotics and high-dose hydrocortisone treatment. CT revealed bilateral juxta-ovarian masses (6.2 x 3.6 x 7.4 cm left and 5 x 2.2 x 3.2 cm right) that on MRI were iso-intense in T1 and hypointense in T2, with early enhancement and rapid washout. One week of high-dose hydrocortisone resulted in significant clinical and laboratory improvement and the patient was discharged with 2 mg dexamethasone/day. One month later US revealed shrinkage of the masses and dexamethasone dose was decreased. At three months from discharge, she has resumed regular menses, and a repeated MRI revealed the para-ovarian masses have shrunk. One year after the diagnosis, the para-ovarian masses have shrunk more to 2.8 x 1.9 x 4.3 on the left and 2.1 x 0.9 x 1.2 on the right with less contrast enhancement in comparison to previous test possibly due to fibrotic changes of the tissue. OARTs are rare tumors with a poorly known natural history, and surgery has been the first option in the few reported cases. We demonstrate that medical treatment is a good alternative, leading to significant tumor shrinkage over a short period.

## INTRODUCTION

The development of gonadal adrenal rest tumors is a benign complication of congenital adrenal hyperplasia (CAH). They probably derive from adrenal cells that migrate with the gonad during fetal development (1). Their growth is usually attributed to poor medical control, but in some cases, the pathogenesis is less clear (2). Adrenal rests are more common in males (testicular adrenal rest tumors, TARTs) and rarely persist into adulthood. Less than 20 cases of ovarian adrenal rest tumors (OARTs) have been reported, and all but one surgically treated (Table 1). We describe a case of a young woman with uncontrolled classical CAH who presented with bilateral para-OARTs that were successfully treated with steroid replacement therapy.

**Table 1:** OARTs cases reported in the literature

Year (Ref.)	Diagnosis	Presenting age	Location	Diagnostic modality	Size	Treatment
1973 (17)	CAH	8 yrs	Ovarian	Arteriography	8cm	Surgery
1979 (15)	Nelson Syndr.	35 yrs	Para-ovarian	Venous sampling	Multiple	Surgery
1982 (18)	Nelson Syndr.	49 yrs	Para-ovarian	CT / scan negative	2cm	Surgery
1986 (13)	Cushing Syndr.	2 yrs	Ovarian	-	10cm	Surgery
1991 (19)	CAH 11-OHD	26 yrs	Ovarian/Leydig Tu	-	-	Surgery
1994 (20)	CAH-HSD3B2	41 yrs	Ovarian/para-aortic	-	Multiple	Surgery
1998 (21)	CAH	15 yrs	Ovarian	US	Multiple	Biopsy/steroids
2000 (16)	CAH 11-OHD	8.5 yrs	Ovarian	US non-diagnostic	2.5cm	Surgery
2001 (14)	CAH 21-OHD	36 yrs	Ovarian	CT	2.8cm	Surgery
2006 (22)	CAH	0.3 yrs	Ovarian	-	Multiple	Post-mortem
2010 (24)	CAH	18 yrs	Ovarian	PET FDG	-	Surgery
2013 (23)	CAH 21-OHD	17 yrs	Ovarian	US / MRI	5cm	Surgery
2014 (25)	CAH 21-OHD	17 yrs	Ovarian	MRI	3+1.7cm	Surgery
2017 (12)	CAH 21-OHD	9 yrs	Ovarian	US/MRI non-diagnostic	Multiple	Surgery
	CAH 21-OHD	15 yrs	Ovarian	US/MRI non-diagnostic	Multiple	Surgery
	CAH 21-OHD	9 yrs	Ovarian	US/MRI non-diagnostic	Multiple	Surgery
(26) 2018	CAH 21-OHD	23 yrs	Para-ovarian	US / CT	6+5cm	Surgery
Present	CAH 21-OHD	20 yrs	Para-ovarian	US / CT / MRI	7cm	Medical

## CASE REPORT

A 20-year-old woman presented to the emergency room with severe abdominal pain, vomiting, diarrhea, and fever. She was known to have salt losing classical CAH treated with mineralocorticoids (fludrocortisone 0.1) and glucocorticoids (10 mg twice daily or 3 times daily alternately). Biochemical data leading to her diagnosis of salt-losing classical CAH at birth were lacking. Moreover, virilization caused clitoromegaly and congenital labial adhesion, separated by urologic surgery at the age of 3. The patient’s menarche was at 13 years old, and she was graded Tanner Stage 5 and had hirsutism (Ferimman-Galley 16) and acne. She reached a final height of 161 cm.

According to genetic testing carried out at diagnosis, she was a heterozygous carrier of the V281L mutation of the *CYP21* gene. Genetic screening was carried out many years ago, with only a few mutations found (cluster E6, Q318X, V281L, I2 splice, I172N, P30L, 8bP del). A deletion of the second allele could not be ruled out given the assays available at the time. Since the test did not correlate with the severity of the phenotype, the patient was advised to repeat genetic counseling several times as follow-up but did not proceed with the tests. A phenotype-genotype discordance was found in previous studies exploring the spectrum of disease severity in CAH, and although rarely, V281L mutation has been associated with salt-losing CAH (when sequencing of the entire gene was not performed) (3).

**Figure 1:**
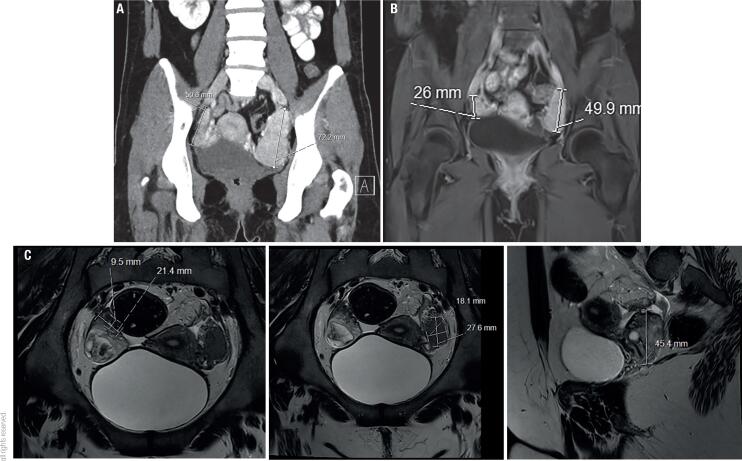
(**A**) CT scan at admission showing bilateral para-ovarian masses) 62 x 36 x 72 mm on the left, 56 x 22 x 32 mm on the right). (**B**) MRI scans 3 months post-admission showing as isointense in mass T1 and hypointense in T2, with early enhancement and rapid washout and significant shrinkage of the masses. (**C**) MRI scan 1-year post-admission showing shrinkage of the masses.

As a result of the patient’s poor compliance, six months prior to her presentation, the hirsutism worsened and amenorrhea, hyperpigmentation, and weakness developed. On admission, she had orthostatic hypotension, diffuse abdominal tenderness, and hyperpigmentation of the skin, mainly of the palms, nipples, and face. Laboratory results revealed leukocytosis of 17.4 k/uL (4-11), hyponatremia of 131 mmol/L (135-145), and elevated C-reactive protein (CRP) levels (343 mg/L). Adrenocorticotropic hormone (ACTH) level was > 278 pmol/L (1.1-10.1), consistent with very poor compliance, and the 17-hydroxyprogesterone (17OHP) level was 91.3 nmol/L (follicular phase 0.9-2.7, luteal phase 0.9-7.57). Parenteral antibiotics and a high-dose hydrocortisone (100 mg/3 daily) treatment were initiated, though no specific pathogen was documented in the blood or urine. A computed tomography (CT) scan (Figure 1) revealed large bilateral para-ovarian masses measuring 4.6 x 3.3 x 7 cm on the left and 2.5 x 1.6 x 3.9 cm on the right; the adrenal glands were also enlarged, especially the left one. Magnetic resonance imaging (MRI) showed the masses were isointense in T1 and hypointense in T2, with early enhancement and rapid washout. A transabdominal ultrasound found the same masses, with no evidence of ovarian torsion. After one week of 300 mg of hydrocortisone daily, significant clinical and laboratory improvements were seen, and a multidisciplinary team decided to continue the conservative approach. The patient was discharged with 2 mg dexamethasone once daily and fludrocortisone 0.1 mg. One month later, US revealed shrinkage of the masses, and the dexamethasone dose was decreased to 1 mg/day.

At three months from discharge, we found a decrease in androgens and ACTH levels (Table 2), she had resumed regular menses, and a repeated MRI (Figure 1) revealed the para-ovarian masses had shrunk to 2.7 x 2.4 x 5 cm on the left and 1.6 x 1.4 x 2.3 cm on the right. Dexamethasone levels were decreased to 0.25 mg/day. It is worth noting that before presentation, the patient lost 20 kg over several months. Upon admission, the patient was underweight (weight: 45 kg,BMI: 17.5). During the treatment with high-dose steroids, her weight increased to 64 kg (her regular weight), but the following year, her weight increased to and remained stable at 80 kg. She had striae on her arms and abdomen, and for a brief period, blood tests showed impaired fasting glucose levels.

**Table 2:** Androgen levels during follow up

	At presentation	3 months after presentation	1 year after presentation	Reference values
ACTH (pmol/L)	>278	249	8.95	1.1-10
17OH-P (nmol/L)	91	1.86	20	0.9-2.7
A (nmol/L)	9.28	2.5	10	0.7-3.1
DHEA-S (µmol/L)	>27	4.99	25	3.92-10.66
T (nmol/L)	8.3	1.3	2.6	0.3 and 2.4

ACTH: adrenocorticotropic hormone; 17-OH-P: 17-hydroxyprogesterone; A: androstenedione; DHEA-S: dehydroepiandrosterone sulfate; T: testosterone.

One year after the diagnosis, the para-ovarian masses had shrunk to 2.8 x 1.9 x 4.3 on the left (down from 4.6 x 3.3 x 7 cm) and 2.1 x 0.9 x 1.2 on the right (down from 2.5 x 1.6 x 3.9) with less contrast enhancement in comparison to the previous test, possibly due to fibrotic changes of the tissue. In addition, multiple enlarged follicles were seen in both ovaries, implying hyperstimulation, possibly secondary to the long-lasting ovarian depression. Oral contraceptives were added to the pharmaceutical regimen.

## DISCUSSION

TARTs are benign tumors that histologically and functionally resemble adrenal tissue and are suspected to arise from aberrant adrenal cells that descend during fetal life with the gonads (4). They are the main cause of infertility in male patients with CAH due to obstruction and compression of the seminiferous tubules (5). The incidence of TARTs varies considerably (0-94%) between series depending on the patient sample and diagnosis modality. TARTs can develop in prepubertal children, especially those with poorly controlled adrenal disease (6). Guidelines recommend sonographic screening beginning at adolescence and every 1-2 years thereafter (7). Adrenal rests are already present in embryonic life. Hence, their prevention is probably impossible (4). Treatment with glucocorticoids can achieve tumor shrinkage, though in some patients, even over suppression might not be effective (8).

In contrast to TARTS in CAH males, OARTs are very rare tumors, and their location in the para-ovarian space has been poorly described. A possible explanation for this gender difference may lie in embryological differences. During the development of the primordial sex cords, adjacent and aberrant adrenal cells can easily migrate and nestle. They will later become the rete testis and seminiferous tubules. In the female embryo, the primary sex cords regress (together with any aberrant adrenal cells nestled in them) and secondary sex cords develop (5). WNT4, a locally acting cell signal, plays an active key role in the development of the female embryo by regulating Mullerian duct formation, controlling steroidogenesis in the gonad, and possibly supporting oocyte development (9). WNT4 inhibits steroidogenic cell migration from the mesonephros into the female gonad. The *Wnt4* gene is downregulated in males to allow for the coelomic vessel formation in the testis; hence, steroidogenic cell migration remains possible (10).

The imaging findings in TARTs are well described (11). The sonographic appearance is of intratesticular hypoechoic lesions, and in an MRI, they are isointense on T1- and hypointense on T2-weighted imaging and show enhancement after intravenous contrast. In a group of 13 females with treated CAH, OARTs were not detected by MRI or US according to TARTs diagnostic criteria (12). Interestingly, OARTs in a Chinese case series presented with small (0.5 cm) nodules discovered during surgery and not detected by imaging. (13).

Clinically significant OARTs have been reported mostly as case studies. No guidelines for the diagnosis or management of OARTs exists. We found only 17 case reports of OARTS in English language literature (13-27), documented in patients with CAH and various enzymatic deficiencies and patients with Nelson syndrome after bilateral adrenalectomy due to Cushing’s disease (Table 1). Poor adherence to treatment and highly elevated ACTH levels were hallmarks of these cases, all but one of which were treated with surgery. In one instance, biopsies were taken with no surgical excision, followed by successful conservative management (22). Since the trigger for most CAH cases was poor adherence to medical treatment, thought should be given to optimizing surveillance and making the transition from pediatric to adult care gradually (7). To the best of our knowledge, ours is the first case in which large para-ovarian OARTs were conservatively and successfully managed based on clinical grounds rather than surgical pathology. Close follow-up is needed in these cases to ensure adherence to therapy.
